# How equitable are community health worker programmes and which programme features influence equity of community health worker services? A systematic review

**DOI:** 10.1186/s12889-016-3043-8

**Published:** 2016-05-20

**Authors:** Rosalind McCollum, Woedem Gomez, Sally Theobald, Miriam Taegtmeyer

**Affiliations:** Liverpool School of Tropical Medicine, Pembroke Place, Liverpool, L3 5QA UK

**Keywords:** Equity, Inequity, Community health worker, Close-to-community provider, Systematic review

## Abstract

**Background:**

Community health workers (CHWs) are uniquely placed to link communities with the health system, playing a role in improving the reach of health systems and bringing health services closer to hard-to-reach and marginalised groups. A systematic review was conducted to determine the extent of equity of CHW programmes and to identify intervention design factors which influence equity of health outcomes.

**Methods:**

In accordance with our published protocol, we systematically searched eight databases from 2004 to 2014 for quantitative and qualitative studies which assessed access, utilisation, quality or community empowerment following introduction of a CHW programme according to equity stratifiers (place of residence, gender, socio-economic position and disability). Thirty four papers met inclusion criteria. A thematic framework was applied and data extracted and managed, prior to charting and thematic analysis.

**Results:**

To our knowledge this is the first systematic review that describes the extent of equity within CHW programmes and identifies CHW intervention design features which influence equity. CHW programmes were found to promote equity of access and utilisation for community health by reducing inequities relating to place of residence, gender, education and socio-economic position. CHWs can also contribute towards more equitable uptake of referrals at health facility level. There was no clear evidence for equitable quality of services provided by CHWs and limited information regarding the role of the CHW in generating community empowerment to respond to social determinants of health. Factors promoting greater equity of CHW services include recruitment of most poor community members as CHWs, close proximity of services to households, pre-existing social relationship with CHW, provision of home-based services, free service delivery, targeting of poor households, strengthened referral to facility, sensitisation and mobilisation of community. However, if CHW programmes are not well planned some of the barriers faced by clients at health facility level can replicate at community level.

**Conclusions:**

CHWs promote equitable access to health promotion, disease prevention and use of curative services at household level. However, care must be taken by policymakers and implementers to take into account factors which can influence the equity of services during planning and implementation of CHW programmes.

**Electronic supplementary material:**

The online version of this article (doi:10.1186/s12889-016-3043-8) contains supplementary material, which is available to authorized users.

## Background

There have been substantial global reductions in child and maternal mortality over the past two decades [1]. However, dramatic differences in mortality and life expectancy exist between and within countries [2, 3]. Evidence has consistently shown that disadvantaged groups have poorer survival chances [2, 4] and lower use of facility-based services [5]. As the World Health Organization ‘Closing the Gap in a Generation’ report (2008) describes ‘gender, education, occupation, income, ethnicity and place of residence are all closely linked to people’s access to, experiences of, and benefits from health care’ (page 8 [2]).

Global interest and investment in community health services has been building to address these gaps and as a pathway to Universal Health Coverage (UHC), with substantial commitment to Community Health Worker (CHW) programmes (see key definitions) in resource-constrained health systems [7–9]. Work of CHWs has been shown to improve equitable child survival, health and nutrition [1, 10] by bringing services closer to the homes of hard-to-reach and underserved populations [6, 11, 12]. The effectiveness of using CHWs to promote immunisation and initiation of breastfeeding and to reduce maternal and child morbidity and mortality, compared with usual care has been demonstrated [13]. However, new health interventions typically reach those with higher socio-economic position first, only benefiting the poor later, in what is known as the ‘inverse equity hypothesis’ [14] and so introducing CHWs within a health system should not be assumed to automatically result in equitable coverage of health services [15]. A wide range of intervention design factors that may be inequitably applied influence CHW performance, such as a mix of incentives, frequent supervision, continuous training, community involvement and strong coordination between CHWs and health workers [11, 16]. Community, economic, socio-cultural factors and education status of the target group (among other factors) have also been demonstrated to influence CHW performance and service coverage [17]. There is a need to better understand the design and contextual factors of CHW programmes which impact health equity within populations.

We conducted a systematic review that followed the Preferred Reporting Items for Systematic Reviews and Meta-Analysis Equity (PRISMA-E) guidelines as these were specifically designed to help reviewers identify, extract, and synthesise evidence on equity in systematic reviews [18]. We set out to respond to two research questions:

1. What evidence is there of (in)equity in CHW programmes?
2. What influences how equitable CHW programmes are in terms of access, utilisation, quality and community empowerment?

## Methods

In this section we summarise the development of guiding conceptual framework, key definitions, search strategy development, selection criteria, data quality assessment, data extraction and data synthesis and analysis.

### Conceptual framework

**Fig. 1:**
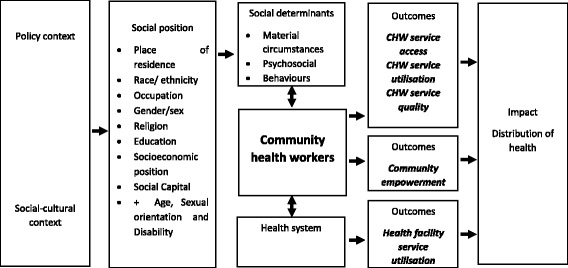
Conceptual framework for factors influencing equity of services provided by community health workers (CHWs). Outcomes in italics, indicate key areas of investigation for this review

We developed a conceptual framework (Fig. 1) derived from three complementary pieces of work:

1. An initial reading of the literature relating to equity and service provision [2, 4, 19–23] led to the identification of the importance of policy and context in influencing social position which in turn influences the four key outcomes—access, utilisation, quality and community empowerment.
2. An adaptation of the PROGRESS plus^1^ framework developed for ensuring explicit consideration of equity in systematic reviews and new intervention studies [24] led to the inclusion of equity stratifiers defined by social position.
3. Our own previous research [17, 25] revealed the importance of social determinants and the interface role played by CHWs between the communities they live within and their health system.

**Table:** 

Key Definitions CHW: Any health worker carrying out functions related to health care delivery; trained in some way in the context of the intervention, and having no formal professional or paraprofessional certificate or degree in tertiary education (page 7 [13]). Equitable CHW programme: CHW services contribute towards eliminating unnecessary and avoidable differences in health, where the whole population has equal access to CHW services with appropriate uptake of referral to health facility according to need, utilisation of CHW services according to need and equal quality of CHW services for all [4] contributing towards community empowerment to tackle underlying social determinants of health, so that everyone can attain their full health potential. Access to CHW services: The delivery of community health services in a timely manner within the client’s home or community, including coverage of services. Utilisation of CHW services: The acceptance and use of community health services provided by CHW either within the home or a local village health post. Uptake of referral: The acceptance and use of services provided at a health facility following referral by a CHW. Quality of CHW services: The delivery of community health services by a CHW which adhere to an evidence base resulting in improved health outcomes in an efficient manner, with optimal safety for clients and which take into account client preferences and aspirations [64]. Community empowerment: Both individuals and communities are involved in active participation in community health activities by building capacity and confidence in order to address and tackle power and control over their lives [69].

### Definitions and search strategy

In accordance with our published protocol [26] we systematically searched Cochrane Central Register of Controlled Trials (CENTRAL), PUBMED, SCOPUS, Science Direct, Global Health, Social Science Citation Index, CINAHL (for published studies) and POPLINE (for grey literature) to identify suitable studies for inclusion.

Search terms to identify relevant studies included CHW terms, health equity terms (including specific search for terms relating to socioeconomic position, gender, disability and place of residence) and the outcome terms access, utilisation, quality and community empowerment, according to our conceptual framework.^2^

Delimiters were English language and studies published between January 2004 to April 2014, in order to capture the most recent findings working within the time and resources available. The search strategy was developed for use with PUBMED (see Additional file 1), translated and modified for use in the other databases, using controlled vocabulary as appropriate. Reference lists of included papers were searched for potential relevant papers.

### Selection Criteria

Studies were selected for inclusion based on “fitness for purpose” rather than following a hierarchy of evidence, as recommended for equity related reviews [2, 18, 27]. This involved reviewing all relevant articles which met inclusion criteria. Both quantitative and qualitative studies and conference abstracts were selected based on their study objectives and were not excluded based on study methodology.

Inclusion criteria^3^ were: studies which provided an analysis of CHW programme outcome (access, utilisation, quality, empowerment); studies which adopted a universal approach to community health i.e. services provided for an entire population^4^ [28]; studies from high, middle or low income country; any study where CHW programme was conducted at primary/ community level. Exclusion criteria: study published before 2004; non-English language; narratives; opinion pieces or commentaries.

### Data extraction and quality assessment

A coding framework was adapted from the data extraction form used by Kok et al. [11] to reflect the conceptual framework, piloted prior to use and modified through an iterative process following familiarisation with the data. The framework (Additional file 2) was applied and data coded for extraction using NVivo version 10 software. Quality was assessed by applying a modified version of the Critical Appraisal Skills Programme (CASP) quality assessment checklist (Additional file 3) [29].

### Data synthesis

Thematic narrative analysis was used adopting an iterative approach to identify and synthesise concepts found in the studies as a result of the heterogeneity of interventions and outcomes [30]. Narratives were developed and data charted based upon themes arising within the data. Findings for each outcome (access, utilisation, quality, empowerment) and its equity stratifiers were identified as follows:

- Pro-equity = improvement in outcome for vulnerable groups (vulnerable groups based upon PROGRESS plus criteria) compared with the general population / no difference in the outcome between vulnerable groups and the rest of the population
- Anti-equity = vulnerable groups have lower/ deteriorating outcomes compared with general population as a consequence of the CHW programme
- Mixed equity = some improvement in an outcome for a vulnerable group but inequities still persist.

These were summarised and charted to provide an insight into the extent of (in)equity. Findings for utilisation were divided into two main aspects: 1) Acceptance and use of community health services provided by CHW either within the home or at a local village health post or 2) Uptake of referrals made by a CHW to services provided at a health facility. Comparisons were carried out capturing study context and CHW programme design features exploring similarities and differences in explaining the findings [16].

RM reviewed titles and abstracts to assess eligibility for inclusion. RM assessed full texts against inclusion criteria by completion of an inclusion criteria checklist. WG assessed all full texts selected for inclusion plus 10 % of remaining full text articles using the inclusion criteria checklist. RM coded and extracted data and assessed quality. WG reviewed data extraction providing additional inputs if and when required and assessed quality. Any differences between reviewers were resolved through discussion. Persisting disagreements were resolved by seeking a third reviewer’s opinion (MT).

## Results

### What evidence is there of (in)equity in CHW programmes?

**Fig. 2:**
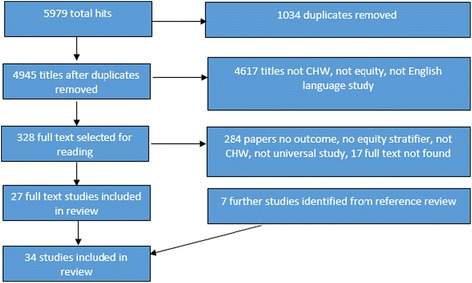
PRISMA Flow chart search results

Overall, we found that despite extensive studies (4945 titles) and after reading 328 full text papers there were limited studies which assess the level of equity of CHW programmes (34 papers included, from 32 studies (Fig. 2 and Table 1)). In total 29 papers were quantitative and five were mixed method papers. Of the papers included (see Table 1) 11 provided an equity analysis of the accessibility of services, 29 of the utilisation of services (26 for utilisation of CHW services provided within the community and six for uptake of referral by CHW to health facility services), five an analysis of the quality of services and five for community empowerment. Papers were identified as low (5), medium (11) or high (18) quality using the CASP checklist, see Table 1 for quality assessment and study designs.

**Table 1:** summarising country, type of CHW and service provided for included studies

Article and Country	Type of CHW	CHW intervention	Study design and overall quality	Equity Stratifier
(Atkinson & Haran, 2005); Brazil	Community Health Worker	Comprehensive family health programme, including CHW component (not well explained)	Cross-sectional household survey; High	Quality – Place of residence -
(Baqui et al., 2009); Bangladesh	Community Health Worker	Maternal and Neonatal health programme with CHW treatment for neonatal infections in intervention areas	Cluster randomised controlled trial; High	Access – Socio-economic status (SES) +
				Utilisation – SES +
				Utilisation – Education +
(Baqui et al., 2008); India	Community Health Worker	NGO facilitation of government Maternal and child health programme	Controlled before and after study; High	Empowerment – SES +
(Bell, Go, Miguel, Parks, & Bryan, 2005); Philippines	Village health worker	Malaria control and case management, community education and bednet distribution	Cross-sectional household survey (including villages with and without resident VHW); Medium	Access - Place of residence -
				Access – Education –
				Access – SES-
				Utilisation – Place of residence –
				Utilisation – Gender +
				Utilisation – Education +
				Utilisation – Social capital -
(Callaghan-Koru et al., 2013); Malawi	Health Surveillance Assistant	Maternal and newborn health programme, including antenatal and postnatal home visits by HSA	Before and after study, with no comparison; High	Access – SES -
				Utilisation – SES ?
				Empowerment – SES +
(Dalal et al., 2013) Kenya	Counsellor	Home Based Testing and Counselling Programme	Longitudinal study; Medium	Utilisation – Gender -
				Utilisation – Age +
				Utilisation – Place of residence -
(DasGupta, Mansuri, Nistha, & Vishwanath, 2007) Pakistan	Lady health worker	Maternal and neonatal health programme offering health and family planning services	Cross-sectional study (used data from Pakistan Integrated Household Survey); Low	Access – Education -
				Utilisation – Gender +
				Utilisation – Education +
(Fort, Grembowski, Heagerty, Lim, & Mercer, 2012) Guatemala	Community Nurse Auxilliary	Comprehensive family health programme	Longitudinal prospective cohort; Medium	Utilisation – Language +
				Utilisation- Education ?
				Utilisation – Place of residence -
				Utilisation – risk +
				Utilisation – SES +
				Utilisation – Age
				Utilisation – Religion –
				Utilisation – Family type –
				Utilisation – Occupation +
				Quality – Age -
				Quality – Language +
				Quality – Education +
(Fylkesnes et al., 2013); Zambia	Counsellor	Home Based Testing and Counselling programme	Cluster randomised controlled trial; High	Utilisation – Education +
				Utilisation – Gender +
				Utilisation – Age +
(Hasegawa, Yasuoka, Ly, Nguon, & Jimba, 2013); Cambodia	Village malaria worker	Child health programme providing malaria case management and child health services	Cross-sectional study; High	Utilisation – Place of residence +
				(- those over 25 km away)
				Utilisation – SES +
				Utilisation – Education +
				Utilisation – Age +
				Utilisation – Occupation +
(Helleringer, Kohler, Frimpong, & Mkandawire, 2009); Malawi	Counsellor	Home Based Testing and Counselling survey	Cross-sectional study; Medium	Utilisation – SES +
				Utilisation – Age +
				Utilisation – Gender -
				Utilisation – Marital status -
				Utilisation – Education -
(Hossain, Khuda, & Phillips, 2004) Bangladesh	Female Welfare Assistant	Family Planning programme	Retrospective re-analysis of longitudinal data; Low	Access – Place of residence – Quality-
(Kamiya, Yoshimura, & Islam, 2013) Bangladesh	Family Welfare Volunteer	Maternal and neonatal health programme, including community mobilisation through community support groups	Controlled, non-randomised before and after study; High	Utilisation - SES + (non CHW-)
	Self Help Group			
(Katabarwa et al., 2010); Uganda	Community distributors	Onchocerciasis control using kinship enhanced delivery model	Controlled cross-sectional study (kinship vs non-kinship); High	Access – Social capital -
				Quality – Social capital –
				Empowerment – Social capital +
(Kisia et al., 2012) Kenya	Community Health Worker	Malaria case management for children under 5 years	Before and after study, no comparison; High	Utilisation – SES +
				Utilisation – Education +
				Utilisation – Village size -
				Utilisation – Age +
				Utilisation – Household size +
(Littrell, Moukam, Libite, Youmba, & Baugh, 2013) Cameroon	Community Health Worker	Community Case Management for children under 5 years	Quasi-experimental study, with comparison group; High	Utilisation – SES +
(Matovu et al., 2005); Uganda	Counsellor	Home Based Testing and Counselling survey	Cross-sectional survey; Medium	Utilisation – Age +
				Utilisation – Education +
				Utilisation - Gender +
				Utilisation – Marital status +
(Anthony K Mbonye, Bygbjerg, & Magnussen, 2007); Uganda^a^	Mixed	Intermittent presumptive treatment malaria in pregnancy provided by a range of community based providers	Before after with comparison	Utilisation – Age
			Qualitative study; High	Utilisation – Place of residence
				Utilisation – Education +
(A K Mbonye, Schultz Hansen, Bygbjerg, & Magnussen, 2008) Uganda ^a^	Mixed	Intermittent presumptive treatment malaria in pregnancy	Before after with comparison; Medium	Utilisation – Age -
				Utilisation – Education +
				Utilisation – Occupation +
				Utilisation – Place of residence +
				Utilisation – Marital status +
(Mukanga et al., 2012); Uganda	Community Health Worker	Community Case Management for pneumonia and fever	Cross-sectional survey; High	Access – Place of residence +
				Utilisation – Place of residence +
				Utilisation - Education - +
				Utilisation – SES +
				Utilisation – Occupation +
(Mulogo, Abdulaziz, Guerra, & Baine, 2011) Uganda	Counsellor	Home Based Testing and Counselling	Longitudinal study with cross sectional and investigative phases; High	Utilisation – Gender –
				Utilisation – Education +
				Utilisation – Place of residence +
				Utilisation – Marital status –
				Utilisation – SES -
(Mumtaz et al., 2013); Pakistan	Lady health worker	Maternal and child health programme providing door step family planning, antenatal and child health services	Cross-sectional study	Access – Social capital -
			Qualitative study; High	Access – SES +
				Quality – Social capital -
(Mutale, Michelo, Jürgensen, & Fylkesnes, 2010) Zambia	Counsellor	Home Based Testing and Counselling	Cross-sectional study; Medium	Utilisation – Place of residence +
				Utilisation – Gender +
				Utilisation – Education +
				Access – Age +
(Naik, Tabana, Doherty, Zembe, & Jackson, 2012); South Africa	Counsellor	Home Based Testing and Counselling	Cluster randomised trial with comparison, comparing home based HTC with facility based; High	Utilisation – Gender -
				Utilisation – Age +
(Nsungwa-Sabiiti et al., 2007); Uganda	Drug distributor	Malaria case management and malaria counselling	Quasi-experimental before after study with comparison group; Medium	Utilisation – SES -
				Utilisation –Gender +
				Utilisation – Education +
(Onwujekwe, Ojukwu, Shu, & Uzochukwu, 2007) Nigeria	Community Health Worker	Malaria case management	Before after study, no comparison; Medium	Access – SES –
				Access – Number household residents –
				Access – Age -
				Utilisation – SES -
				Quality – SES -
(Perry, King-Schultz, Aftab, & Bryant, 2007); Haiti	Animatrice	General health programme involving household peer to peer education	Cross-sectional study	Access – Place of residence –
	Matrons		Exit interview; Low	
	Health Agents			
	Monitrices			
(Quayyum et al., 2013); Bangladesh	Shasthaya Shebika Shasthya Kormi	Maternal and neonatal health programme providing maternal health services and education at home	Quasi-experimental, before after study with comparison area; High	Utilisation – SES + (non CHW +/-)
	Newborn Health workers			
(Quinley & Govindasamy, 2007); Nepal	Female Community Health Volunteer	Child health (no details provided)	Cross-sectional study (additional analysis of Demographic Health Survey data); Low	Utilisation – SES -
				Utilisation – Place of residence +
(Siekmans et al., 2013); Kenya	Community Health Worker	Malaria case management for under fives	Before after study, no comparison area; High	Access – SES +
				Utilisation – SES +
				Empowerment – SES +
(D. O. Simba, 2005) Tanzania ^b^	Community based distributor	Family Planning provision of contraceptives and information of sexual and reproductive health	Descriptive cross-sectional study; Medium	Utilisation – Age -
				Utilisation – Occupation +
				Utilisation - Gender +
				Utilisation – Religion +
				Utilisation – SES +
				Quality – SES –
				Empowerment – SES +
(D. Simba, Schuemer, Forrester, & Hiza, 2011); Tanzania^b^	Community Based Agent	Family Planning provision of contraceptives and information of sexual and reproductive health	Cross-sectional descriptive study; Low	Utilisation – Place of residence +
				Quality – SES +
(Wolff et al., 2005) Uganda	Counsellor	Home Based Testing and Counselling	Repeated cross-sectional study	Utilisation – Age +
			Qualitative study; Medium	Utilisation – Gender +
(Wringe et al., 2008); Tanzania	Counsellor	Voluntary Counselling and Testing offered at purpose built hut following household questionnaire	Repeated cross-sectional study; High	Utilisation – Gender -
				Utilisation – Education -
				Utilisation – Religion -
				Utilisation – Race -
				Utilisation – Place of residence –
				Utilisation – Age -

Note + pro equity, - anti equity, ? mixed equity findings

^a^Indicates two papers based on the same study

^b^Indicates two papers based on the same study

There was a notable difference in the content of intervention packages between continents. Papers from the Americas (3) presented findings from comprehensive family health programmes; papers from Asia (10) focused on a particular population group, for example maternal and newborn health and papers from Africa (21) tended to have a more disease specific focus, such as malaria or HIV. This difference in the comprehensiveness of CHW programmes in itself raises equity questions, particularly within the African context.

Our findings reveal that CHW interventions adopting a universal approach can result in improved equity for CHW service access and use (see Table 1 and Additional file 4: Tables S2–S4). CHW services were found to reduce inequities relating to access for place of residence and socio-economic position.

Acceptance and use of community health services provided by CHWs either within the home or local village health post was reported in 26 studies (Additional file 4: Table S3). In some studies CHW services reduced inequities according to place of residence, gender, education, socio-economic position, age, religion, occupation and marital status for community level services. CHW programmes also have the potential to contribute to more equitable uptake of referrals for health facility services by reducing barriers due to socio-economic position, language and risk identified through six studies (Additional file 4: Table S4).

Quality was less frequently described despite being an important dimension of equity, with only five studies reporting findings for quality. Studies mainly assessed quality in terms of satisfaction from the patient’s perspective. Findings for quality tended to be negative, with no clear evidence for equitable quality of CHW services (Additional file 4: Table S5).

Findings from five studies (Additional file 4: Table S6) indicate that CHW programmes can generate some degree of community empowerment by utilising existing social capital and addressing knowledge gap according to socio-economic position.

### What influences how equitable CHW programmes are in terms of access, utilisation, quality and community empowerment?

Our review identified that the same supply side and demand side barriers which limit equity of health services delivered from health facility can also influence the equity of CHW programmes if not adequately addressed during planning (see Table 2). This will be further expounded in addressing the second study question.

**Table 2:** indicating how CHW interventions can overcome supply and demand side barriers to equity

Barrier	How CHW intervention can overcome barrier	Equity considerations for CHW programme planners
*Supply side (CHW services)*		
Low number of health workers in hard-to-reach areas	Local recruitment of CHWs, including recruitment of CHWs from marginalised groups	Ensure CHWs are recruited locally, not centrally
		Consider options to include illiterate CHWs in areas where education levels are low
		Ensure CHW selection reflects community – inclusion of CHWs from marginalised groups
Time taken to reach service location	Provision of services within the client’s home	CHW intervention planning to consider geographic features – reduced household numbers per CHW where households are far apart/ difficult terrain
Cost of services	Free service provision	Payment for services can continue to present a barrier to service use, even if CHW services are provided within the home
*Demand side (CHW services)*		
Demand for services and information about health care	Developing improved client knowledge about CHW role as health care providers through home visits, sensitisation meetings and community mobilisation	Consider comprehensive package of services, rather than single disease specific intervention
		Weak sensitisation and community mobilisation around CHW intervention can lead to limited demand for services
		Consider alternative approaches for certain groups – e.g. HTC provision by a non-resident CHW for youth and work based HTC (rather than home based) for migrant men
Waiting time for services, indirect costs (transport), opportunity costs	Provision of curative services and provision of HTC within the home	Ensure strong supply chain for commodities to all CHWs
		Need for supportive supervision
		Need for strong referral links between community and health facility
Education	Reducing the knowledge/ behaviour gap between richest and poorest community members through one-to-one and group education	Need to plan for behaviour change communication within CHW programme design
Household expectations and community and cultural preferences	Provision of services within the home in cultural contexts where women are reluctant to seek care outside their home.	Need for consideration of existing social relationships between clients and CHW
*Demand side (Health facility services)*		
Demand for services and information about health care	CHW led demand creation strategies, community engagement and action planning	Consider the package of services provided at community level and whether this could reduce use of services by skilled provider at health facility (e.g. ANC)
	CHW training in problem solving	
	Use of a household risk assessment by CHW to ensure high risk households receive more frequent home visits to advise about for clinic attendance	
Waiting time for services, indirect costs (transport), opportunity costs	Reimbursement for transportation	Transport and opportunity costs will still exist, even where community is empowered and so community funds/ transport refunds are useful tools to overcome this barrier
	Community funds	
Education	Reducing the influence of education on health facility service utilisation among those with limited formal education through one-to-one and group education	Failure to develop community empowerment through support groups may hinder use of services at health facility level
Household expectations and community and cultural preferences	CHW accompaniment during referrals	Consider incentive for CHW to refer and accompany clients to health facility

#### Factors affecting accessibility

Proximity of the service to the household is a vital factor in reducing inequities relating to place of residence [31]. However, when the CHW was not resident within the community [32] or intervention design did not vary the ratio of the number of households to the number of CHWs for different geographic areas (mountain versus plain), population dispersion [6] or intensity of tasks required of CHWs [33] inequities persisted with those living further from the CHW less likely to receive household visit [6, 33].

Pre-requisite educational requirements within certain CHW programmes [32, 34] resulted in more CHWs being recruited from and operating within communities with higher educational levels, thereby putting illiterate communities at a disadvantage.

Home visits by CHWs were more common among the most poor in three studies due to a range of reasons such as non-governmental organisation (NGO) facilitation with supervision and monitoring [5], recruitment of the most poor as CHWs [35] and deliberate targeting of most poor households for services by CHWs [36]. However, payments (for malaria treatment) remained a barrier to CHW service access for the most poor [37]. Recruitment of CHWs from areas outside their catchment community in Malawi was found to result in fewer household visits for the most poor as CHWs were less likely to reside in more remote areas [38].

Social capital (defined as the presence of an existing family, kin or social relationship with the CHW) was influential in three studies with CHWs more likely to provide home visits or services to those with whom they have a kinship or a pre-established relationship [32, 35, 39]. This can have positive sequelae if the CHW is preferentially recruited from a poorer household, as was the case in Pakistan where the CHW role is considered low status and so attracts poorer women [35]. These findings are summarised in Additional file 4: Table S2. 

#### Factors affecting utilisation

**Fig. 3:**
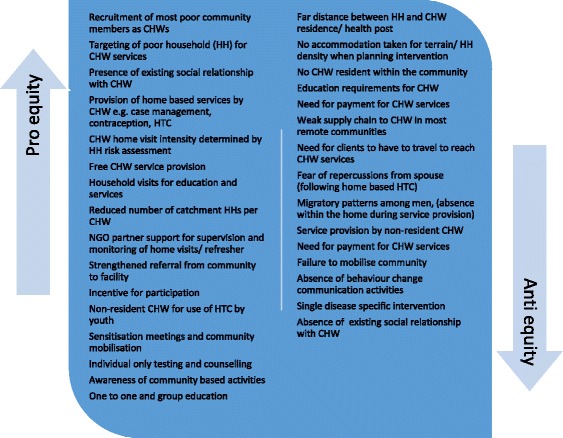
Key CHW intervention features promoting or threatening equity of CHW service access, use, quality and community empowerment

**Fig. 4:**
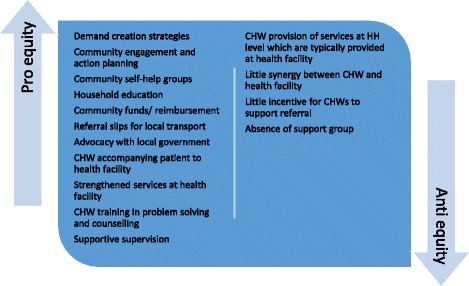
CHW intervention features promoting or threatening equitable uptake of CHW referral to health facility services

Findings for utilisation are divided into two main aspects: 1) Acceptance and use of community health services provided by CHW either within the home or at a local village health post (see Fig. 3) or 2) Uptake of referrals made by a CHW to services provided at a health facility (see Fig. 4).

1. *Acceptance and use of community health services*

Those living further from the health facility were found to be more likely to use CHW services in five studies where the intervention included home visits by CHW [40]; CHW provision of case management [31, 41]; contraception [42] or home based HIV testing and counselling (HTC) [43, 44]. However, poor supply chain and lack of CHW supplies was found to result in lower CHW utilisation rates for in one study for those living farthest (over 25 km) from the health facility [41]. Meanwhile, geographic barriers continued to persist where clients had to travel to CHW home/health post in four studies with lower service utilisation for those living farthest from the CHW home/ health post [31, 32, 45] and higher utilisation in a roadside community [46] and for the urban population compared with rural [47].

Use of CHW services was demonstrated to reduce gender differences or to have no difference in utilisation rates between men and women in eight studies (four non- HTC studies and four home based HTC). Provision of services by CHWs within the home in Pakistan helped to alleviate gender constraints which women face in using maternal child health services in a context where women’s movements are restricted [34]. However, gender inequities still persisted in five HTC studies, with fear of repercussions from spouses constraining women from disclosing sexually transmitted infection (STI) related symptoms during home based HTC compared with facility based [44] and higher utilisation of home based HTC among women in four studies, thought to be due to migratory patterns among men [48]. Where HTC was provided by CHW at clinic or purpose-built hut rather than being home based, utilisation was more common among men [46]. Women described difficulty finding time to leave the home and the need for standards of dress at health facility [49]. These gender inequities were virtually eliminated following home based delivery of HTC [49].

Utilisation was found to be higher among the least educated in three studies, influence of education was reduced following CHW provision of services within the home in four papers and no influence of education on CHW service utilisation in seven studies, although limited explanation was provided. However, where CHW services were provided by non-resident CHW [50] or at a purpose built hut [46] service utilisation remained lower among the least educated.

CHWs strongly promoted more equitable utilisation of services according to socio-economic position (Additional file 4: Table S3), with nine papers reporting that utilisation of CHW services was more common among the poorest compared with the least poor within the population. Common features of CHW programmes which are utilised more by the poorest include free services; community based distribution of contraceptives; household visits for health education, HTC and curative services and community level case management. Other features of these CHW programmes include reduced number of catchment households for CHW [51]; NGO partner support through supervision and/ or refresher [36, 51–54]; strengthened referral from community to facility [51] and incentives for participation [50]. However, some inequities still persisted with three studies reporting that CHW service utilisation was less common among the poorest. Barriers to use of CHW services by the poorest included need for payment for malaria treatment in Nigeria [37] or free service delivery, but failure to mobilise mothers [55]; absence of behaviour change communication activities [55]; single disease specific (malaria) CHW intervention [37, 55].

Four studies described that provision of services by CHWs resulted in increased utilisation among previously underserved age groups, particularly HTC among youth. Features of these CHW programmes include non-resident CHW [43, 47, 50]; sensitisation meetings or community mobilisation in advance [47, 50] and individual only HTC [47, 50]. However, five studies reported no effect and two reported that use of CHW services was lower among selected age groups (youth) due to fear that home based HTC would prompt speculation from family members [49].

Utilisation of CHW services was also influenced by the presence of pre-existing social connections to CHW, religion, race, occupation and marital status. These findings are summarised in Additional file 4: Table S3.

2) *Uptake of referrals made by a CHW to services provided at a health facility*.

Socio-economic status was the leading influential stratifier for the uptake of referral (described in five studies). Increased utilisation of health facility services among the poor compared with the less poor in two studies [51, 56] included specific strategies to increase demand for services; community engagement through development of action plans involving both facility and community stakeholders; establishment of community support self-help groups to support women from low socio-economic position households; household education; community funds/ financial support/reimbursement; referral slips for local transport; advocacy for maternal health with local government; CHW accompanying patients to facility and strengthened services at facility level. However, use of CHW referral to health facility level services increased among the poor but still remained skewed in favour of the less poor in two studies [5, 38]. Common features of these studies included demand generation through home visits, training of CHWs in problem solving and counselling skills; supportive supervision; NGO support; CHW assessment and referral if danger signs present; establishment of demand generation groups. Use of health facility level services actually reduced among the poorest in one study due to the provision of antenatal care (ANC) by trained CHW at household level [51]. There was no improvement in uptake of facility level services in two studies due to little synergy between the CHW and the health facility, with little incentive for CHWs to support or facilitate referral to the health facility [34] or where there was no participant support group [56].

Level of risk influenced utilisation of health facility services in Guatemala, with higher use of health facility level services among households with higher risk and who spoke only one language. In this study the CHW carried out a household risk assessment, which identified households as high, medium or low risk and frequency of household visit varied accordingly, with households identified as high risk receiving most frequent CHW visits which encouraged use of services at health facility as needed. The study intervention also included support from supervision and referral team [45]. These findings are summarised in Additional file 4: Table S4.

#### Factors affecting quality

There were no studies that identified quality indicators. Perceived quality, using satisfaction with services as a proxy for quality was associated with awareness and practice of community based activities. Satisfaction was found to be higher in rural areas compared with urban [57] in one study in Brazil. The reason for higher satisfaction in rural areas was unclear but may relate to social, economic and cultural differences or differing expectations by populations. Two studies found lower quality for the poorest clients with lower reported satisfaction [58] and use of lower quality treatment by the poorest compared with the richest, where clients had to pay CHWs for malaria treatment [37]. Meanwhile having a pre-existing kinship relationship with the CHW resulted in greater satisfaction in two studies [35, 39]. These findings are summarised in Additional file 4: Table S5.

#### Factors affecting community empowerment

Studies mainly assessed community empowerment in terms of knowledge/behaviour change following CHW programme and indicate that CHW programmes can generate some degree of community empowerment by reducing the knowledge gap between richest and poorest in four studies which included a CHW role for health education via one-to-one and group education [58] and home visits [5, 38]. Meanwhile, community ownership was higher where a kinship relationship pre-existed with the CHW. This was shown to result in greater community involvement in deciding the location of the treatment centre, CHW selection and mobilisation and more respondents having received health education in kinship enhanced compared with no pre-existing kinship relationship with the CHW [39]. These findings are summarised in Additional file 4: Table S6. 

## Discussion

Our literature review adds to the evidence base for CHWs by providing the first review describing the extent of equity within different CHW programmes in different contexts and describing the features of CHW programmes which influence equity. The limited focus and evidence on equity that we found is in keeping with a recent review of maternal and child interventions on equity [30]. This highlights the evaporation of equity from policy (which often introduces CHW programmes as a means to promote both universal health coverage and equity [59]) to practice, where there is limited ongoing monitoring and evaluation of equity indicators within CHW programme design, in keeping with evidence for building equitable health systems [60]. This systematic review is therefore a timely review of current evidence, highlighting research gaps and priorities for future research.

### Supply and demand barriers and equity following introduction of CHW programmes

This systematic review has found that CHWs are able to address both supply side barriers (aspects of health systems that hinder service uptake) and demand side barriers (factors influencing the ability to use health services at individual, household or community level [12]) to uptake of health services. However, it is important that policy makers consider design features which may hinder equity when planning programmes (see Table 2). These include supply side barriers such as low numbers of health workers (including CHWs), time to reach service, cost of services [12, 61] and demand side barriers to both CHW services and uptake of health facility services, such as demand for services and information about health care, waiting time, indirect and opportunity costs, education, household expectations, community and cultural preferences [12, 62].

### Quality and equity following introduction of CHW programmes

This review has highlighted gaps in the extent to which quality is monitored with regards to equity stratifiers. Quality is widely identified as being a central tenet to equity [2, 4, 63]. It would therefore be expected that the quality of services provided by CHWs would be evaluated during studies which monitored equity. However, this review revealed quality was assessed in only five studies, four of which evaluated client satisfaction and one of which assessed use of more effective versus less effective anti-malarial treatment provided by CHW [37]. None of the included studies assessed the technical quality of services provided by the CHW according to any equity stratifier. This finding reveals a disparity between this review’s findings and the literature on quality of care, which includes equity as one of the six dimensions of quality health care (effective, efficient, accessible, acceptable/ patient centred, equitable and safe) [64]. For CHW programmes to ensure quality equitable service provision for all groups it is vital that quality improvement (QI) approaches (which measure and understand performance gaps, before introducing, monitoring and evaluating interventions to close these gaps [65]) include an equity focus. Various tools for quality improvement have already been created, such as the CHW assessment and improvement matrix (CHW AIM) [66] and the authors would propose that equity be added as an additional programmatic component to this tool to ensure regular and consistent application of an equity lens during QI approaches for CHW programmes.

### Community empowerment and equity following introduction of CHW programmes

It is commonly acknowledged that underlying social determinants must be addressed in order to tackle underlying causes of health inequities [2, 63]. CHWs are uniquely positioned as a link between the community and the health system. CHWs live within communities where they work and are able to observe these social determinants for health during household visits and day-to-day interactions with other members of their communities. However, while a recent literature review identified evidence for the empowerment of a CHW through his/her work, there was limited evidence described within the review regarding empowerment of the community through the CHW for implementation of lay health worker programmes [16]. Our systematic review found minimal literature which assesses the role of CHWs in tackling social determinants for health, which is in keeping with findings presented in Collaboration for Applied Health Research and Delivery (CAHRD) discussion paper during CAHRD consultation 2014 [67]. The role of CHWs as agents for change working within their communities to identify and address social determinants for health is a key and vital gap in the literature which the authors feel should be addressed through future implementation research.

### Strengths and weaknesses of the data

Given, the extent and volume of published literature about CHWs, there were limited studies which assessed the equity of these CHW programmes where a universal approach was adopted. Community health is being adopted widely as a means to promote and ensure universal health coverage and to build resilience [7–9, 68]. It is therefore vital that adequate research assesses the equity of these services and identifies factors which influence provision of community health service delivery using a universal approach, in order to ensure community health services are planned and implemented in the most equitable manner. CHW intervention planning should take into account differences between contexts and measure service access, utilisation, quality and community empowerment for marginalised groups.

A notable omission is data relating to the equity of services for people with disabilities. Despite this being included within the search strategy, none of the studies included within this review provided evidence regarding the equity of services for people with disability as part of a CHW programme adopting a universal approach. There was also a lack of data regarding equity of services for older populations, as much of the age related data referred to youth (although age was not included as a specific search term). There is therefore an urgent need to better understand equity of CHW services for people with disabilities and older age groups.

### Study limitations

We attempted to include a range of study designs in order to capture useful information and a range of search engines were used, including POPLINE, which sought to capture some unpublished studies. However, seven papers were identified from additional sources, rather than through the original search and so it is possible that some studies may not have been published which may have included relevant information. Due to the current emphasis of using CHW programmes as a means of increasing universal health coverage, we determined to include studies which adopted a universal approach, in order to elucidate findings applicable for CHW programmes applied to whole populations. However, there were a number of studies which were excluded based on having adopted a targeted approach rather than universal, as it is difficult to truly assess equity using targeted studies, but they may have had included valuable lessons.

Assessment of quality was conducted to provide greater understanding for the reader of the quality of data included within the review, but was not used to ‘weigh’ synthesis of evidence for included studies.

## Conclusion

We found that CHW programmes across diverse contexts promote more equitable access and use of CHW services at household level and have the potential to contribute towards improved uptake of referral for health facility services. However, care must be taken by policymakers and implementers to take into account CHW programme features which can influence the equity of services provided during planning and implementation of CHW programmes. The quality of CHW services for differing socio-demographic groups and the role of CHWs in empowering communities to address underlying social determinants for change are key gaps in the current CHW evidence base. It is vital that equity indicators are included within routine CHW monitoring and that equity is incorporated within quality improvement approaches for community health to ensure that the pro-equity statements in CHW policies do not evaporate in practice. We recommend that evidence based decision-making by policymakers take into consideration the underlying programme features which influence the equity of CHW interventions in addition to performance (motivation and competencies); effectiveness and cost-effectiveness.

### Ethics approval and consent to participate

Since it is a review paper, the study did not need ethical committee approval.

### Consent for publication

Not applicable as manuscript does not contain any individual persons data.

### Availability of data and materials

Protocol for this review was registered with PROSPERO and is available at http://www.crd.york.ac.uk/PROSPERO/DisplayPDF.php?ID=CRD42014013067.

References for included papers are included within list of references.

## Additional files

Additional file 1: Equity and community health workers search strategy. (DOCX 28 kb) Additional file 2: Coding Framework. (DOCX 11 kb) Additional file 3: Modified Critical Appraisal Skills Programme (CASP). **Table S1A** Modified CASP Checklist. **Table S1B** Example completion of Modified CASP checklist. (DOCX 14 kb) Additional file 4: Table S2. Summarising equity findings for access of CHW services. **Table S3** Summarising utilisation of CHW services at household or health post and equity. **Table S4** summarising uptake of referral by CHW for health facility services. **Table S5 **Summarising quality of CHW services and equity. Table S6 summarising community empowerment. (DOCX 107 kb)

## Abbreviations

CASP: Critical Appraisal Skills Programme
CHW: Community Health Worker
CTC: Close-To-Community
HTC: HIV Testing and Counselling
IPTp: Intermittent preventive treatment in pregnancy
NGO: Non Governmental Organization
PROGRESS: Place of residence, race, occupation, gender, religion, education, socioeconomic status, social capital
SES: Socio Economic Status
UHC: Universal Health Coverage
WHO: World Health Organization

## Footnotes

1 PROGRESS plus is an acronym referring to: place of residence; race; occupation; gender; religion; education; socioeconomic position; social capital; plus – disability; age; sexual orientation.

2 Identified papers which included findings for additional stratifiers were analysed for all PROGRESS plus equity stratifiers.

3 CHW inclusion and exclusion criteria are identified in Additional file 1.

4 Given that the purpose of the review is to identify lessons for policymakers implementing universal CHW programs, it is difficult to truly assess equity through a targeted study and so only studies with a universal approach were included.
