# Supplementary material for: How equitable are community health worker programmes and which programme features influence equity of community health worker services? A systematic review
Source: BMC Public Health. 2016 May 20;16:419. doi: 10.1186/s12889-016-3043-8 (PMC4875684; doi:10.1186/s12889-016-3043-8)
Supplement: Additional file 1: — Equity and community health workers search strategy. (DOCX 28 kb) [file 12889_2016_3043_MOESM1_ESM.docx]

**Additional file 1: Equity and community health workers search strategy**

**Community health worker definition**

We defined a CHW as ‘any health worker carrying out functions related to health care delivery; trained in some way in the context of the intervention, and having no formal professional or paraprofessional certificate or degree in tertiary education’ (page 7 [10]). This CHW definition falls within the broader group of close-to-community providers, ‘who are health workers forming the first point of contact at community level, having up to 3 years paraprofessional training’ [8]. Our CHW definition for the purposes of this review excluded formally trained workers, CHW with tertiary education, interventions performed as an extension of the participant’s profession, e.g. teachers conducting health promotion in schools, patient support groups, peer counselling in schools, intervention where CHW was trained to deliver services only to his/her own family, intervention where CHW component can’t be disentangled from other health systems interventions, short term campaigns (e.g. vaccination campaigns), untrained traditional birth attendant/ informal provider.

This search strategy has been developed based upon search terms for lay health worker developed by Lewin et al. (2010) and subsequently modified by Kok et al. (2013). The search terms used by Kok et al. (2013) formed the basis of this community health worker search strategy (although auxiliary search terms added in addition to the Lewin (2010) search strategy were not included since the definition of lay health worker adopted follows that described by Lewin et al. (2010)).

Search terms for equity and equity outcomes have been developed based upon reading of related literature. Relevant search terms were developed and MeSH terms exploded for Scopus, Web of Science, Global Health and CINAHL.

Modified shorter versions were adopted for use with Sciencedirect and Popline due to limitations on the length of search string.

**SCOPUS SEARCH**

| Scopus  Filters   - Year 2004 – 2014 - English language - Abstract   Search #1 and #2 and #3 |
| --- |

COMMUNITY HEALTH WORKER TERMS

| #1 | "community health worker" OR "community health workers" OR "community health aides" OR "community health aide" OR "family planning personnel" OR "village health worker" OR "village health workers" OR "home health aide" OR "home health aides" OR "allied health personnel" OR "population program specialists" OR "nurses aides" OR "nurses aide" OR "nursing auxiliaries" OR "nursing auxiliary" OR "volunteerism" OR "non professional home care" OR "nonprofessional home care" OR "peer group" OR "social support" OR "psychosocial networks" OR "social networks" OR "lay worker" OR "lay workers" OR "lay visitors" OR "lay attendants" OR "lay support" OR "lay person" OR "lay personnel" OR "lay helpers" OR "lay carer" OR "lay carers" OR "lay caregiver" OR "lay caregivers" OR "lay staff" OR "lay midwife" OR "lay midwives" OR "lay providers" OR "lay health worker" OR "lay health workers" OR "lay counsellor" OR "lay counsellors" OR "lay volunteer" OR "lay volunteers" OR "lay mentor" OR "lay mentors" OR "voluntary workers" OR "voluntary worker" OR "voluntary visitor" OR "voluntary visitors" OR "voluntary support" OR "voluntary supporter" OR "voluntary supporters" OR "volunteer worker" OR "volunteer workers" OR "volunteer support" OR "volunteer supporters" OR "volunteer supporter" OR "volunteer caregivers" OR "volunteer caregiver" OR "volunteer staff" OR "volunteer providers" OR "volunteer provider" OR "volunteer care givers" OR "volunteer practitioners" OR "volunteer care" OR "volunteer nursing" OR "informal workers" OR "informal visitor" OR "informal visitors" OR "informal support" OR "informal supporter" OR "informal supporters" OR "informal helpers" OR "informal carer" OR "informal carers" OR "informal caregiver" OR "informal caregivers" OR "informal providers" OR "informal care givers" OR "informal practitioners" OR "untrained workers" OR "untrained attendants" OR "untrained person" OR "untrained personnel" OR "untrained staff" OR "untrained midwives" OR "untrained providers" OR "untrained practitioners" OR "Unlicensed visitor" OR "unlicensed visitors" OR "unlicensed support" OR "unlicensed supporters" OR "unlicensed supporter" OR "unlicensed personnel" OR "unlicensed caregivers" OR "unlicensed staff" OR "unlicensed providers" OR "unlicensed practitioners" OR "nonprofessional workers" OR "non professional workers" OR "non professional support" OR "nonprofessional supporter" OR "nonprofessional supporters" OR "nonprofessional personnel" OR "non professional personnel" OR "non professional carers" OR "nonprofessional caregivers" OR "non professional caregivers" OR "nonprofessional staff" OR "non professional staff" OR "non professional visitor" OR "non professional visitors" OR "non professional providers" OR Paraprofessional* OR paramedic OR paramedics OR "paramedical worker" OR "paramedical workers" OR "paramedical personnel" OR "allied health worker" OR "allied health workers" OR "support worker" OR "support workers" OR "Trained volunteer" OR "trained volunteers" OR "trained health worker" OR "trained health workers" OR "trained mothers" OR "trained health care workers" OR "trained healthcare workers" OR "community health care workers" OR "community healthcare workers" OR "community distributors" OR "community based health workers" OR "community based distributors" OR "village healthcare workers" OR "village health workers" OR "village health care workers" OR "community based providers" OR "community workers" OR "community based workers" OR "frontline health workers" OR "frontline health worker" OR "frontline workers" OR "frontline worker" OR "community volunteer" OR "community volunteers" OR "community based volunteers" OR "community support" OR "community based support" OR "birth attendant" OR "birth attendants" OR "birth assistants" OR Doula* OR douladural* OR Monitrice* OR "peer volunteer" OR "peer volunteers" OR "peer mentor" OR "peer mentors" OR "peer support" OR "peer intervention" OR "peer interventions" OR "peer counsellor" OR "peer counsellors" OR "church based intervention" OR "church based interventions" OR "church based program" OR Linkworker OR linkworkers OR "link worker" OR "link workers" OR "barefoot doctor" OR "barefoot doctors" OR Outreach OR "home care" OR "home aide" OR "home aides" OR "home nursing" OR "home support" OR "home intervention" OR "home interventions" OR "home treatment" OR "home treatments" OR "home visitor" OR "home visitors" OR "expert patient" OR "expert patients" OR "health promoter" OR "health promoters" OR "health extension worker" OR "health extension workers" OR "mentor mother" OR "mentor mothers" |
| --- | --- |

EQUITY TERMS

| #2 | "rural population" OR "rural populations" OR "rural community" OR "rural communities" OR "rural spatial distribution" OR "healthcare disparities" OR "healthcare disparity" OR "health care disparity" OR "health care disparities" OR Disadvantage* OR "vulnerable population" OR "vulnerable populations" OR "underserved patients" OR "underserved population" OR "underserved populations" OR "sensitive population groups" OR "sensitive population group" OR "sensitive populations" OR "sensitive population" OR "medically underserved area" OR "physician shortage area" OR Inequalit* OR "socioeconomic factors" OR "socioeconomic factor" OR "low income population" OR "low income populations" OR "standard of living" OR "gender identity" or Gender OR "sex role" OR "woman role" OR "women role" OR "man role" OR "men role" OR "gender role" OR equit* OR inequit* or disparit* OR equalit* OR disabilit* |
| --- | --- |

OUTCOME TERMS

| #3 | "health services accessibility" OR "access to health care" OR "accessibility of health services" OR "health services geographic accessibility" OR "contraceptive availability" OR "program accessibility" OR "program availability" OR "availability of health services" OR "health services availability" OR "acceptability of health care" OR "acceptability of healthcare" OR "patient acceptance of health care" OR "patient acceptance of healthcare" OR "program acceptability" OR "health care seeking behaviour" OR "health seeking behaviour" OR "patient acceptance of healthcare" OR "acceptors" OR "acceptor characteristics" OR "community empowerment" OR "utilization" OR "coverage" OR "community governance" OR "quality of healthcare" OR "quality of health care" OR "healthcare quality" OR "health care quality" |
| --- | --- |

**PUBMED SEARCH**

| Pubmed  Additional filters   - Searched in title/ abstract - English - Last 10 years   Search #1 AND #2 AND #3 |
| --- |

COMMUNITY HEALTH WORKER TERMS

| #1 | ((((((((((community health workers[MeSH Terms]) OR home health aides[MeSH Terms]) OR allied health personnel[MeSH Terms]) OR aides, nurses'[MeSH Terms]) OR voluntary workers[MeSH Terms]) OR home nursing[MeSH Terms]) OR peer groups[MeSH Terms]) OR social support[MeSH Terms])) OR (("lay worker" OR "lay workers" OR "lay visitors" OR "lay attendants" OR lay support* OR lay person* OR "lay helpers" OR "lay carer" OR "lay carers" OR "lay caregiver" OR "lay caregivers" OR "lay staff" OR "lay midwife" OR "lay midwives" OR "lay providers" OR "lay health worker" OR "lay health workers" OR lay counsel* OR "lay volunteer" OR "lay volunteers" OR lay mentor* OR "voluntary workers" OR "voluntary visitor" OR "voluntary visitors" OR voluntary support* OR "volunteer worker" OR "volunteer workers" OR volunteer support* OR "volunteer caregivers" OR "volunteer staff" OR "volunteer providers" OR "volunteer care givers" OR "volunteer practitioners" OR "volunteer care" OR "volunteer nursing" OR "informal workers" OR informal visit* OR informal support* OR "informal helpers" OR "informal carer" OR "informal carers" OR "informal caregiver" OR "informal caregivers" OR "informal providers" OR "informal care givers" OR "informal practitioners" OR "untrained workers" OR "untrained attendants" OR untrained person* OR "untrained staff" OR "untrained midwives" OR "untrained providers" OR "untrained practitioners" OR Unlicensed visit* OR unlicensed support* OR unlicensed person* OR "unlicensed caregivers" OR "unlicensed staff" OR "unlicensed providers" OR "unlicensed practitioners" OR "nonprofessional workers" OR "non-professional workers" OR non-professional support* OR nonprofessional person* OR non-professional person* OR "non-professional carers" OR "nonprofessional caregivers" OR "non-professional caregivers" OR "nonprofessional staff" OR "non-professional staff" OR non-professional visit* OR "non-professional providers" OR Paraprofessional* OR paramedic OR paramedics OR "paramedical worker" OR "paramedical workers" OR "paramedical personnel" OR "allied health worker" OR "allied health workers" OR "support worker" OR "support workers" OR "Trained volunteer" OR "trained volunteers" OR "trained health worker" OR "trained health workers" OR "trained mothers" OR "trained health care workers" OR "trained healthcare workers" OR "community health care workers" OR "community healthcare workers" OR "community distributors" OR "community-based health workers" OR "community-based distributors" OR "community health aides" OR "village healthcare workers" OR "village health workers" OR "village health care workers" OR "community-based providers" OR "community workers" OR "community-based workers" OR "frontline health workers" OR "frontline health worker" OR "frontline workers" OR "frontline worker" OR "community volunteer" OR "community volunteers" OR "community-based volunteers" OR "community support" OR "community-based support" OR "birth attendant" OR "birth attendants" OR "birth assistants" OR Doula* OR douladural* OR Monitrice* OR "peer volunteer" OR "peer volunteers" OR peer mentor* OR "peer support" OR "peer intervention" OR "peer interventions" OR peer counsel* OR "church based intervention" OR "church based interventions" OR church based program* OR Linkworker OR linkworkers OR "link worker" OR "link workers" OR "barefoot doctor" OR "barefoot doctors" OR Outreach OR "home care" OR "home aide" OR "home aides" OR "home nursing" OR "home support" OR "home intervention" OR "home interventions" OR "home treatment" OR "home treatments" OR home visit* OR "expert patient" OR "expert patients" OR "health promoter" OR "health promoters" OR "health extension worker" OR "health extension workers" OR "mentor mother" OR "mentor mothers"))) |
| --- | --- |

EQUITY TERMS

| #2 | ((((((rural populations[MeSH Terms]) OR health care disparities[MeSH Terms] OR factors, socioeconomic[MeSH Terms]) OR disadvantaged[MeSH Terms]) OR medically underserved areas[MeSH Terms]) OR vulnerable populations[MeSH Terms]) OR inequality[MeSH Terms]) OR gender[MeSH Terms] OR (equit* OR inequit* or disparit* OR equalit* OR inequalit* OR disabilit*) |
| --- | --- |

OUTCOME TERMS

| #3 | ((((((((((health services accessibility[MeSH Terms]) OR health services availability[MeSH Terms]) OR acceptability of health care[MeSH Terms]) healthcare quality[MeSH Terms]) OR ("community empowerment" OR utilization OR coverage OR "community governance" OR "health care seeking behaviour" or "health seeking behaviour")))))))) |
| --- | --- |

**COCHRANE CENTRAL SEARCH**

| Cochrane Central  No additional filters added |
| --- |

#1 MeSH descriptor: [Community Health Workers] explode all trees

#2 MeSH descriptor: [Home Health Aides] explode all trees

#3 MeSH descriptor: [Allied Health Personnel] explode all trees

#4 MeSH descriptor: [Nurses' Aides] explode all trees

#5 MeSH descriptor: [Volunteers] explode all trees

#6 MeSH descriptor: [Peer Group] explode all trees

#7 MeSH descriptor: [Social Support] explode all trees

#8 (lay or voluntary or volunteer or volunteers or untrained or unlicensed or non-professionals or non-professional or nonprofessionals or nonprofessional or "non professional" or "non professionals" or informal or "non formal" or "non-formal") near/5 (worker or workers or visitor or visitors or attendant or attendants or aide or aides or support or support* or person* or person or helper or helpers or carer or carers or caregiver or caregivers or consultant or consultants or assistant or assistants or staff or visit* or visit or midwife or midwives or provider or providers or "care giver" or "care givers" or practitioner or practitioners)

#9 Paraprofessional or paraprofessionals or paramedic or paramedics or "paramedical worker" or "paramedical workers" or "paramedical personnel" or "allied health worker" or "allied health workers" or "support worker" or "support workers"

#10 trained near/3 (volunteer or volunteers or "health worker" or "health workers" or mother or mothers)

#11 (community or communities or "community based" or village or villages or frontline) near/3 ("health worker" or "health workers" or "health care worker" or "health care workers" or "healthcare worker" or "healthcare workers" or distributor or distributors or worker or workers or provider or providers)

#12 (community or communities or "community based") near/3 (volunteer or volunteers or aide or aides or support)

#13 (birth or childbirth or labor or labour) next/1 (attendant or attendants or assistant or assistants)

#14 Monitrice or monitrices or doula or douladural*

#15 (lay or peer) next/1 (volunteer or volunteers or mentor* or mentor or counsel* or support or intervention or interventions)

#16 "church based" near/3 (intervention or interventions or program* or program or counsel*)

#17 linkworker or linkworkers or "link worker" or "link workers"

#18 "barefoot doctor" or "barefoot doctors"

#19 Outreach

#20 home next/1 (care or aide or aides or nursing or support or intervention or interventions or treatment or treatments or visit* or visit)

#21 (care or aide or aides or nursing or support or intervention or interventions or treatment or treatments or visit* or visit) near/3 (lay or volunteer or volunteers or voluntary)

#22 "expert patient" or "expert patients" or "health promoter" or "health promoters" or "health extension worker" or "health extension workers" or "mentor mother" or "mentor mothers"

#23 #20 and #21

#24 #1 or #2 or #3 or #4 or #5 or #6 or #7 or #8 or #9 or #10 or #11 or #12 or #13 or #14 or #15 or #16 or #17 or #18 or #19 or #22 or #23

#25 MeSH descriptor: [Rural Population] explode all trees

#26 MeSH descriptor: [Healthcare Disparities] explode all trees

#27 MeSH descriptor: [Socioeconomic Factors] explode all trees

#28 MeSH descriptor: [Vulnerable Populations] explode all trees

#29 MeSH descriptor: [Medically Underserved Area] explode all trees

#30 MeSH descriptor: [Gender Identity] explode all trees

#31 equit* or inequit* or disparit* or equalit* or inequalit* or disabilit*

#32 #25 or #26 or #27 or #28 or #29 or #30 or #31

#33 MeSH descriptor: [Health Services Accessibility] explode all trees

#34 MeSH descriptor: [Patient Acceptance of Health Care] explode all trees

#35 MeSH descriptor: [Quality of Health Care] explode all trees

#36 "community empowerment" or utilization or coverage or "community governance" or "health care seeking behaviour" or "health seeking behaviour"

#37 #33 or #34 or #35 or #36

#38 #24 and #32 and #37

**WEB OF SCIENCE, CINAHL and GLOBAL HEALTH SEARCHES**

| Web of science  Filters   - Year 2004 – 2014 - English language - Core collection - Topic   Search #1 AND #2 AND #3 |
| --- |
| CINAHL searched through University of Liverpool Discover  Filters   - Year 2004 – 2014 - Abstract   Search #1 AND #2 AND #3 |
| Global Health searched through University of Liverpool Discover  Filters   - Year 2004 – 2014 - Abstract   Search #1 AND #2 AND #3 |

COMMUNITY HEALTH WORKER TERMS

| #1 | "community health worker" OR "community health workers" OR "community health aides" OR "community health aide" OR "family planning personnel" OR "village health worker" OR "village health workers" OR "home health aide" OR "home health aides" OR "allied health personnel" OR "population program specialists" OR "nurses aides" OR "nurses aide" OR "nursing auxiliaries" OR "nursing auxiliary" OR "volunteerism" OR "non professional home care" OR "nonprofessional home care" OR "peer group" OR "social support" OR "psychosocial networks" OR "social networks" OR "lay worker" OR "lay workers" OR "lay visitors" OR "lay attendants" OR "lay support" OR "lay person" OR "lay personnel" OR "lay helpers" OR "lay carer" OR "lay carers" OR "lay caregiver" OR "lay caregivers" OR "lay staff" OR "lay midwife" OR "lay midwives" OR "lay providers" OR "lay health worker" OR "lay health workers" OR "lay counsellor" OR "lay counsellors" OR "lay volunteer" OR "lay volunteers" OR "lay mentor" OR "lay mentors" OR "voluntary workers" OR "voluntary worker" OR "voluntary visitor" OR "voluntary visitors" OR "voluntary support" OR "voluntary supporter" OR "voluntary supporters" OR "volunteer worker" OR "volunteer workers" OR "volunteer support" OR "volunteer supporters" OR "volunteer supporter" OR "volunteer caregivers" OR "volunteer caregiver" OR "volunteer staff" OR "volunteer providers" OR "volunteer provider" OR "volunteer care givers" OR "volunteer practitioners" OR "volunteer care" OR "volunteer nursing" OR "informal workers" OR "informal visitor" OR "informal visitors" OR "informal support" OR "informal supporter" OR "informal supporters" OR "informal helpers" OR "informal carer" OR "informal carers" OR "informal caregiver" OR "informal caregivers" OR "informal providers" OR "informal care givers" OR "informal practitioners" OR "untrained workers" OR "untrained attendants" OR "untrained person" OR "untrained personnel" OR "untrained staff" OR "untrained midwives" OR "untrained providers" OR "untrained practitioners" OR "Unlicensed visitor" OR "unlicensed visitors" OR "unlicensed support" OR "unlicensed supporters" OR "unlicensed supporter" OR "unlicensed personnel" OR "unlicensed caregivers" OR "unlicensed staff" OR "unlicensed providers" OR "unlicensed practitioners" OR "nonprofessional workers" OR "non professional workers" OR "non professional support" OR "nonprofessional supporter" OR "nonprofessional supporters" OR "nonprofessional personnel" OR "non professional personnel" OR "non professional carers" OR "nonprofessional caregivers" OR "non professional caregivers" OR "nonprofessional staff" OR "non professional staff" OR "non professional visitor" OR "non professional visitors" OR "non professional providers" OR Paraprofessional* OR paramedic OR paramedics OR "paramedical worker" OR "paramedical workers" OR "paramedical personnel" OR "allied health worker" OR "allied health workers" OR "support worker" OR "support workers" OR "Trained volunteer" OR "trained volunteers" OR "trained health worker" OR "trained health workers" OR "trained mothers" OR "trained health care workers" OR "trained healthcare workers" OR "community health care workers" OR "community healthcare workers" OR "community distributors" OR "community based health workers" OR "community based distributors" OR "village healthcare workers" OR "village health workers" OR "village health care workers" OR "community based providers" OR "community workers" OR "community based workers" OR "frontline health workers" OR "frontline health worker" OR "frontline workers" OR "frontline worker" OR "community volunteer" OR "community volunteers" OR "community based volunteers" OR "community support" OR "community based support" OR "birth attendant" OR "birth attendants" OR "birth assistants" OR Doula* OR douladural* OR Monitrice* OR "peer volunteer" OR "peer volunteers" OR "peer mentor" OR "peer mentors" OR "peer support" OR "peer intervention" OR "peer interventions" OR "peer counsellor" OR "peer counsellors" OR "church based intervention" OR "church based interventions" OR "church based program" OR Linkworker OR linkworkers OR "link worker" OR "link workers" OR "barefoot doctor" OR "barefoot doctors" OR Outreach OR "home care" OR "home aide" OR "home aides" OR "home nursing" OR "home support" OR "home intervention" OR "home interventions" OR "home treatment" OR "home treatments" OR "home visitor" OR "home visitors" OR "expert patient" OR "expert patients" OR "health promoter" OR "health promoters" OR "health extension worker" OR "health extension workers" OR "mentor mother" OR "mentor mothers" |
| --- | --- |

EQUITY TERMS

| #2 | "rural population" OR "rural populations" OR "rural community" OR "rural communities" OR "rural spatial distribution" OR "healthcare disparities" OR "healthcare disparity" OR "health care disparity" OR "health care disparities" OR Disadvantage* OR "vulnerable population" OR "vulnerable populations" OR "underserved patients" OR "underserved population" OR "underserved populations" OR "sensitive population groups" OR "sensitive population group" OR "sensitive populations" OR "sensitive population" OR "medically underserved area" OR "physician shortage area" OR Inequalit* OR "socioeconomic factors" OR "socioeconomic factor" OR "low income population" OR "low income populations" OR "standard of living" OR "gender identity" or Gender OR "sex role" OR "woman role" OR "women role" OR "man role" OR "men role" OR "gender role" OR equit* OR inequit* or disparit* OR equalit* OR disabilit* |
| --- | --- |

OUTCOME TERMS

| #3 | "health services accessibility" OR "access to health care" OR "accessibility of health services" OR "health services geographic accessibility" OR "contraceptive availability" OR "program accessibility" OR "program availability" OR "availability of health services" OR "health services availability" OR "acceptability of health care" OR "acceptability of healthcare" OR "patient acceptance of health care" OR "patient acceptance of healthcare" OR "program acceptability" OR "health care seeking behaviour" OR "health seeking behaviour" OR "patient acceptance of healthcare" OR "acceptors" OR "acceptor characteristics" OR "community empowerment" OR "utilization" OR "coverage" OR "community governance" OR "quality of healthcare" OR "quality of health care" OR "healthcare quality" OR "health care quality" |
| --- | --- |

**POPLINE AND SCIENCEDIRECT SEARCHES**

| Popline  Filters   - English - 2004 – 2014 |
| --- |
| Sciencedirect  Filters   - 2004 – 2014 - All fields |

COMMUNITY HEALTH WORKER TERMS

| #1 | "community health worker" OR "community health aide" OR "family planning personnel" OR "village health worker" OR "home health aide" OR "allied health personnel" OR paramedic* OR "nurses aide" OR nursing auxiliar* OR voluntary worker* OR "untrained personnel" OR "home nursing" OR "peer group" OR "social support" OR "lay worker" OR "informal workers" OR untrained person* OR Unlicensed visit* OR "nonprofessional workers" OR "non-professional workers" OR "allied health worker" OR "support worker" "Trained volunteer" OR "trained mothers" OR "community health care workers" OR "community healthcare workers" OR "community distributors" OR "village health workers" OR "village health care workers" OR "community-based providers" OR "community workers" OR "frontline health workers" OR "community volunteer" OR "birth attendant" OR Doula* OR "peer volunteer" OR "church based intervention" OR Linkworker OR linkworkers OR "link worker" OR "barefoot doctor" OR Outreach OR "home care" OR "home aide" OR "home nursing" OR home visit* OR "expert patient" OR "health promoter" OR "health extension worker" OR "mentor mother" |
| --- | --- |

EQUITY TERMS

| #2 | "rural population" OR "rural populations" OR "rural community" OR "rural communities" OR "rural spatial distribution" OR "healthcare disparities" OR "healthcare disparity" OR "health care disparity" OR "health care disparities" OR Disadvantage* OR "vulnerable population" OR "vulnerable populations" OR "underserved patients" OR "underserved population" OR "underserved populations" OR "sensitive population groups" OR "sensitive population group" OR "sensitive populations" OR "sensitive population" OR "medically underserved area" OR "physician shortage area" OR Inequalit* OR "socioeconomic factors" OR "socioeconomic factor" OR "low income population" OR "low income populations" OR "standard of living" OR "gender identity" or Gender OR "sex role" OR "woman role" OR "women role" OR "man role" OR "men role" OR "gender role" OR equit* OR inequit* or disparit* OR equalit* OR disabilit* |
| --- | --- |

OUTCOME TERMS

| #3 | "health services accessibility" OR "access to health care" OR "accessibility of health services" OR "health services geographic accessibility" OR "contraceptive availability" OR "program accessibility" OR "program availability" OR "availability of health services" OR "health services availability" OR "acceptability of health care" OR "acceptability of healthcare" OR "patient acceptance of health care" OR "patient acceptance of healthcare" OR "program acceptability" OR "health care seeking behaviour" OR "health seeking behaviour" OR "patient acceptance of healthcare" OR "acceptors" OR "acceptor characteristics" OR "community empowerment" OR "utilization" OR "coverage" OR "community governance" OR "quality of healthcare" OR "quality of health care" OR "healthcare quality" OR "health care quality" |
| --- | --- |
