# Supplementary material for: How equitable are community health worker programmes and which programme features influence equity of community health worker services? A systematic review
Source: BMC Public Health. 2016 May 20;16:419. doi: 10.1186/s12889-016-3043-8 (PMC4875684; doi:10.1186/s12889-016-3043-8)
Supplement: Additional file 2: — Coding Framework. (DOCX 11 kb) [file 12889_2016_3043_MOESM2_ESM.docx]

**Additional file 2: Coding Framework**

| **0. Non study related info** |
| --- |
| **1. Study characteristics** |
| 1. 1Background |
| 1.2 Research gaps |
| 1.3 Objective |
| 1.4 Design |
| 1.6 Analysis |
| **2. Setting** |
| 2.1Country |
| 2.2 Equity setting |
| 2.3 Geographical |
| 2.4 Health system |
| 2.5 NGO government |
| 2.5.1.NGO |
| 2.5.2. Government |
| **3. Community health worker** |
| 3.1 Selection and recruitment |
| 3.2 Training |
| 3.3 Tasks |
| 3.4 Management |
| **3a. Intervention** |
| 3a.1 Description |
| 3a.2 Home visitation |
| 3a.3 Self help groups |
| 3a.4 non-CHW |
| **4. Stratifier** |
| 4.1 Place of residence |
| 4.2 Race |
| 4.3 Occupation |
| 4.4 Gender |
| 4.5 Religion |
| 4.6 Education |
| 4.7 Socio economic status |
| 4.8 Social capital |
| 4.9 Disability |
| 4.91 age |
| 4.91 Caste |
| **5. Stakeholders** |
| **6. Outcomes** |
| 6.1 Coverage |
| 6.1.1 Coverage of home visit |
| 6.2 Behaviours |
| 6.2 Utilisation |
| 6.2.1 Health facility service utilisation |
| 6.2.2 Community service utilisation |
| 6.3 Awareness |
| 6.4 Knowledge |
| 6.5 acceptance |
| 6.6 adverse |
| 6.7 quality |
| 6.8 access |
| **7. Explanation** |
| 7.1 Inputs |
| 7.2 Programme implementation |
| 7.3 Intervention target |
| 7.4 Context |
| 7.4.1 geographic |
| 7.4 Socioeconomic context |
| 7.5 Other factors |
| **8. Ongoing research gaps** |
| **9. References** |
