# Supplementary material for: How equitable are community health worker programmes and which programme features influence equity of community health worker services? A systematic review
Source: BMC Public Health. 2016 May 20;16:419. doi: 10.1186/s12889-016-3043-8 (PMC4875684; doi:10.1186/s12889-016-3043-8)
Supplement: Additional file 3: — Modified Critical Appraisal Skills Programme (CASP). Table S1A Modified CASP Checklist. Table S1B Example completion of Modified CASP checklist. (DOCX 14 kb) [file 12889_2016_3043_MOESM3_ESM.docx]

**Additional file 3: Modified Critical Appraisal Skills Programme (CASP)**

Table S1A Modified CASP Checklist

| 1. Is there a clear statement of the aims of the research or the program evaluated? |
| --- |
| 1. Is the study/ program context clearly described? |
| 1. Is the study design or methodology appropriate for the hypotheses/ addressing the aim of the research or program evaluated? |
| 1. Is the recruitment strategy for participants in the study or program appropriate to the aims of the research or the program? |
| 1. Is the method of data collection clearly described and appropriate to the research question/ objective of the evaluation? |
| 1. Is the method of data analysis clearly described and appropriate to the research question/ objective of the evaluation? |
| 1. Are the claims made supported by sufficient evidence? I.e. did the data provide sufficient depth, detail and richness? |
| 6, 7 yes=high quality  4, 5 yes=medium quality  0, 1, 2, 3 yes=low quality |

Table S1B Example completion of Modified CASP checklist

|  | Atkinson et al.,2010 | |
| --- | --- | --- |
|  | Reviewer 1 | Reviewer 2 |
| ·         Is there a clear statement of the aims of the research or the program evaluated? | Y | Y |
| ·         Is the study/ program context clearly described? | N | Y |
| ·         Is the study design or methodology appropriate for the hypotheses/ addressing the aim of the research or program evaluated? | Y | Y |
| ·         Is the recruitment strategy for participants in the study or program appropriate to the aims of the research or the program? | N | Y |
| ·         Is the method of data collection clearly described and appropriate to the research question/ objective of the evaluation? | Y | Y |
| ·         Is the method of data analysis clearly described and appropriate to the research question/ objective of the evaluation? | Y | Y |
| ·         Are the claims made supported by sufficient evidence? I.e. did the data provide sufficient depth, detail and richness? | Y | Y |
| Total yes | 5 | 7 |
| Total no | 2 | 0 |
| Total don’t know | 0 | 0 |
| Consensus post discussion if differing opinion | high | |
