# Supplementary material for: How equitable are community health worker programmes and which programme features influence equity of community health worker services? A systematic review
Source: BMC Public Health. 2016 May 20;16:419. doi: 10.1186/s12889-016-3043-8 (PMC4875684; doi:10.1186/s12889-016-3043-8)
Supplement: Additional file 4: Table S2. — Summarising equity findings for access of CHW services. Table S3 Summarising utilisation of CHW services at household or health post and equity. Table S4 summarising uptake of referral by CHW for health facility services. Table S5 Summarising quality of CHW services and equity. Table S6 summarising community empowerment. (DOCX 107 kb) [file 12889_2016_3043_MOESM4_ESM.docx]

**Additional file 4**

*Table S2 Summarising equity findings for access of CHW services*

| **Equity Stratifier** | **Equity assessment** | **Detail** | **Reference** |
| --- | --- | --- | --- |
| Household proximity to CHW | | | |
| Place of residence | Pro | CHW brings services closer to household | (Mukanga et al., 2012) |
| Place of residence | Anti | Those living further from CHW are less likely to receive household visit/ service | (Bell et al., 2005)  (Hossain et al., 2004)  (Perry et al., 2007) |
| Education of community | | | |
| Education | Anti | Higher level of education in communities with CHW | (Bell et al., 2005)  (DasGupta et al., 2007) |
| Household visit and SES | | | |
| Socio-economic status | Pro | Home visits by CHW more common among the most poor | (Baqui et al., 2008)  (Siekmans et al., 2013)  (Mumtaz et al., 2013) |
| Socio-economic status | Anti | Home visits by CHW more common among the least poor | (Callaghan-Koru et al., 2013) |
| Socio-economic status | Anti | Poor economic status in villages may limit CHW expansion | (Bell et al., 2005) |
| Payment for services and access | | | |
| Socio-economic status | Anti | Poorest have reduced willingness to pay and reduced access to highest cost (in this case also highest quality) services provided by CHW | (Onwujekwe et al., 2007) |
| Service delivery and social capital | | | |
| Social capital | Anti | Home visits/ service provision more common among those with kinship relationship | (Katabarwa et al., 2010)  (Mumtaz et al., 2013)  (Bell et al., 2005) |
| Age and Number of household members | | | |
| Age | Anti | Willingness to pay reduced as age increased | (Onwujekwe et al., 2007) |
| Household size | Anti | Willingness to pay reduced as household size increased | (Onwujekwe et al., 2007) |

Table S3 Summarising utilisation of CHW services at household or health post and equity

| **Equity Stratifier** | **Equity assessment** | **Detail** | **Reference** |
| --- | --- | --- | --- |
| Proximity to health post and CHW utilisation | | | |
| Place of residence | Pro | Those living further from health facility more likely to use CHW services | (Hasegawa et al., 2013)  (A K Mbonye et al., 2008)  (Mukanga et al., 2012)  (Quinley & Govindasamy, 2007)  (D. Simba et al., 2011) |
| Place of residence | Anti | Those living furthest from health facility less likely to use CHW services | (Hasegawa et al., 2013) |
| Proximity to CHW and service utilisation | | | |
| Place of residence | Pro | Place of residence less important as equity stratifier after home based HTC | (Mulogo et al., 2011)  (Mutale et al., 2010) |
| Place of residence | Anti | Utilisation higher among those living in roadside village/ urban setting | (Wringe et al., 2008)  (Dalal et al., 2013) |
| Place of residence | Anti | Service utilisation lowest among those farthest away from CHW home/ post | (Mukanga et al., 2012)  (Fort et al., 2012)  (Bell et al., 2005) |
| Gender and utilisation of CHW services (non-HTC) | | | |
| Gender | Pro | Higher utilisation of CHW services among men (typically underserved for contraception) | (D. O. Simba, 2005) |
| Gender | Pro | Gender no influence on utilisation of CHW services | (Bell et al., 2005)  (Nsungwa-Sabiiti et al., 2007)  (DasGupta et al., 2007) |
| Gender and utilisation of CHW services (HTC) | | | |
| Gender | Pro | Gender inequities reduced following home based HTC | (Wolff et al., 2005)  (Mutale et al., 2010)  (Fylkesnes et al., 2013)  (Matovu et al., 2005) |
| Gender | Anti | Gender inequities persist following home based delivery HTC | (Mulogo et al., 2011) |
| Gender | Anti | Home based HTC Service uptake more common in women | (Naik et al., 2012)  (Dalal et al., 2013)  (Helleringer et al., 2009)  (Mulogo et al., 2011) |
| Gender | Anti | HTC utilisation more common among men | (Wringe et al., 2008) |
| Higher utilisation among lower educated | | | |
| Education | Pro | Service utilisation higher among the least educated | (Baqui et al., 2009)  (Fort et al., 2012)  (Matovu et al., 2005) |
| Education no influence on utilisation | | | |
| Education | Pro | Education less influential after CHW provide services in home | (Mulogo et al., 2011)  (Anthony K Mbonye et al., 2007)  (A K Mbonye et al., 2008)  (Mutale et al., 2010) |
| Education | Pro | Education no influence on utilisation of CHW services | (Fylkesnes et al., 2013a)  (Bell et al., 2005)  (DasGupta et al., 2007)  (Kisia et al., 2012)  (Mukanga et al., 2012)  (Nsungwa-Sabiiti et al., 2007)  (Hasegawa et al., 2013) |
| Lower utilisation among lower educated | | | |
| Education | Anti | HTC CHW service utilisation higher among those with more education | (Wringe et al., 2008)  (Helleringer et al., 2009) |
| Higher utilisation among poorer wealth group | | | |
| Socio-economic status | Pro | Utilisation of services from CHW higher among the poorest | (D. O. Simba, 2005)  (D. Simba et al., 2011)  (Quayyum et al., 2013)  (Baqui et al., 2009)  (Hasegawa et al., 2013)  (Helleringer et al., 2009)  (Kisia et al., 2012)  (Siekmans et al., 2013)  (Littrell et al., 2013) |
| SES no influence on utilisation | | | |
| Socio-economic status | Pro | Socio-economic status no influence on service utilisation | (Fort et al., 2012)  (Mukanga et al., 2012) |
| Lower utilisation among poorer wealth group | | | |
| Socio-economic status | Anti | Utilisation of services from CHW higher among middle income group | (Quinley & Govindasamy, 2007) |
| Socio-economic status | Anti | Service utilisation lower among the poorest | (Nsungwa-Sabiiti et al., 2007)  (Mulogo et al., 2011) |
| Socio-economic status | Anti | Poorest have reduced utilisation of highest cost (in this case also highest quality) services provided by CHW | (Onwujekwe et al., 2007) |
| Higher utilisation among youth | | | |
| Age | Pro | Increased HTC utilisation among younger age groups | (Helleringer et al., 2009)  (Dalal et al., 2013)  (Mutale et al., 2010)  (Wolff et al., 2005) |
| Age | Anti | HTC utilisation lower among older age group | (Wringe et al., 2008) |
| No difference in utilisation according to age | | | |
| Age | Pro | Age no influence on CHW utilisation | (Kisia et al., 2012)  (Matovu et al., 2005)  (Naik et al., 2012)  (Fylkesnes et al., 2013)  (Hasegawa et al., 2013) |
| Lower utilisation among youth | | | |
| Age | Anti | Adolescents less likely to use CHW services | (D. O. Simba, 2005)  (A K Mbonye et al., 2008) |
| Social capital | | | |
| Social capital | Anti | CHW utilisation lower in those with reduced social connections to CHW | (Bell et al., 2005) |
| Religion, race, occupation, language, marital status, village size and utilisation | | | |
| Religion | Anti | HTC utilisation lower among certain religion | (Wringe et al., 2008) |
| Religion | Anti | Those with no religion less likely to use CHW services | (Fort et al., 2012) |
| Religion | Pro | Roman Catholics more likely to access CHW services for contraception | (D. O. Simba, 2005) |
| Race/tribe | Anti | HTC utilisation lower among select tribes | (Wringe et al., 2008) |
| Occupation | Pro | Farmers/unemployed/unskilled more likely to use services than other occupations | (D. O. Simba, 2005)  (A K Mbonye et al., 2008)  (Fort et al., 2012) |
| Occupation | Pro | Occupation no influence on utilisation | (Hasegawa et al., 2013)  (Mukanga et al., 2012) |
| Marital status | Pro | Number of sexual partners no influence on service utilisation | (Matovu et al., 2005) |
| Marital status | Anti | HTC CHW service utilisation lower among those with multiple concurrent partners or never married | (Helleringer et al., 2009)  (Mulogo et al., 2011) |
| Marital status | Pro | CBD use greater among widowed/ separated | (A K Mbonye et al., 2008) |
| Household size | Pro | No difference in CHW utilisation according to household size | (Kisia et al., 2012) |
| Village size | Anti | CHW service utilisation higher in smaller villages | (Kisia et al., 2012) |

Table S4 summarising uptake of referral by CHW for health facility services

| **Equity Stratifier** | **Equity assessment** | **Detail** | **Reference** |
| --- | --- | --- | --- |
| Utilisation of health facility services | | | |
| Socio-economic status | Pro | Utilisation of health facility services increased among the poor | (Kamiya et al., 2013)  (Quayyum et al., 2013) |
| Socio-economic status | Mixed | Utilisation of health facility services increased among the poor but remained skewed in favour of the least poor | (Baqui et al., 2008)  (Callaghan-Koru et al., 2013) |
| Socio-economic status | Anti | Utilisation of some services from skilled health worker reduced as a result of CHW provision | (Quayyum et al., 2013) |
| Socio-economic status | Anti | No improvement in use of health facility services | (Kamiya et al., 2013)  (DasGupta et al., 2007) |
| Family, language, risk | | | |
| Family | Anti | Non-nuclear family less likely to receive antenatal tetanus | (Fort et al., 2012) |
| Language | Pro | Service utilisation higher among those with only 1 spoken language | (Fort et al., 2012) |
| Risk | Pro | Households classified as high risk have higher utilisation rate | (Fort et al., 2012) |

Table S5 Summarising quality of CHW services and equity

| **Equity Stratifier** | **Equity assessment** | **Detail** | **Reference** |
| --- | --- | --- | --- |
| Place of residence, age, language and education and quality of CHW service | | | |
| Place of residence | Anti | Higher satisfaction in rural area | (Atkinson & Haran, 2005) |
| Social capital and satisfaction | | | |
| Social capital | Anti | Satisfaction higher where kinship relationship present | (Katabarwa et al., 2010)  (Mumtaz et al., 2013) |
| SES and quality of CHW service | | | |
| Socio-economic status | Anti | Satisfaction lower for poorest wealth group | (D. O. Simba, 2005) |
| Socio-economic status | Anti | Quality of treatment sought lower for poorest wealth group | (Onwujekwe et al., 2007) |

Table S6 summarising community empowerment

| **Equity Stratifier** | **Equity assessment** | **Detail** | **Reference** |
| --- | --- | --- | --- |
| Knowledge/ behaviour and socio-economic status | | | |
| Socio-economic status | Pro | CHW reduced knowledge/ behaviour gap between richest and poorest | (D. O. Simba, 2005)  (Baqui et al., 2008)  (Callaghan-Koru et al., 2013)  (Siekmans et al., 2013) |
| Community ownership | | | |
| Social capital | Pro | Community ownership policies stronger in kinship enhanced intervention | (Katabarwa et al., 2010) |
